# Cuttlebone-like V_2_O_5_ Nanofibre Scaffolds – Advances in Structuring Cellular Solids

**DOI:** 10.1038/srep42951

**Published:** 2017-02-20

**Authors:** Andrea Knöller, Tomče Runčevski, Robert E. Dinnebier, Joachim Bill, Zaklina Burghard

**Affiliations:** 1 Institute for Materials Science, University of Stuttgart, Heisenbergstr. 3, 70569 Stuttgart, Germany; 2 Department of Chemistry, University of California Berkeley, Berkeley, California 94720, USA; 3 Materials Sciences Division, Lawrence Berkeley National Laboratory, Berkeley, California 94720, USA; 4 Max Planck Institute for Solid State Research, Heisenbergstr. 1, 70569 Stuttgart, Germany

## Abstract

The synthesis of ceramic materials combining high porosity and permeability with good mechanical stability is challenging, as optimising the latter requires compromises regarding the first two properties. Nonetheless, significant progress can be made in this direction by taking advantage of the structural design principles evolved by nature. Natural cellular solids achieve good mechanical stability *via* a defined hierarchical organisation of the building blocks they are composed of. Here, we report the first synthetic, ceramic-based scaffold whose architecture closely mimics that of cuttlebone –a structural biomaterial whose porosity exceeds that of most other natural cellular solids, whilst preserving an excellent mechanical strength. The nanostructured, single-component scaffold, obtained by ice-templated assembly of V_2_O_5_ nanofibres, features a highly sophisticated and elaborate architecture of equally spaced lamellas, which are regularly connected by pillars as lamella support. It displays an unprecedented porosity of 99.8 %, complemented by an enhanced mechanical stability. This novel bioinspired, functional material not only displays mechanical characteristics similar to natural cuttlebone, but the multifunctionality of the V_2_O_5_ nanofibres also renders possible applications, including catalysts, sensors and electrodes for energy storage.

Progress in energy storage and conversion, sensing, filtering, gas distribution and catalysis depends on the availability of functional materials that combine high surface area and permeability into high open porosity, coupled with good mechanical stability^1^,^2^. Porous ceramic materials that fulfil these criteria are accessible through mimicking structuring concepts found in biomaterials^3^,^4^. With a remarkable porosity of 93 %, natural cuttlebone^5^ outperforms most cellular biomaterials, including bone^6^,^7^ (<79 %) and wood^8^ (<70 %). This rigid and ultralight aragonite-based scaffold, found in cuttlefish (*Sepia Officinalis L.*), features a high porosity paired with an excellent mechanical stability, and hence represents an ideal model for the design of advanced, bioinspired functional materials. Like other structural biomaterials, cuttlebone accomplishes its excellent mechanical stability *via* a hierarchically organized structure from the nano- to the micrometre scale. Aragonite fibres of different length, size and orientation^9^, complemented by about 4.5 wt.% of organic phase^5^, form regularly stacked cavities in the form of micrometre-thick lamellas. These lamellas are separated and supported by numerous, evenly distributed, micrometre-thick pillars^10^, resulting in a highly complex porous architecture that is able to resist external pressures of about 1 MPa^5^.

Mimicking the intricate architecture of cuttlebone to obtain highly porous and mechanically stable functional material requires an appropriate substitute for the aragonite fibres as well as an adequate structuring procedure. Based on our previous results^11^, close-to-ideal candidates in this respect are ribbon-like V_2_O_5_ nanofibres – unique one-dimensional oxide nanostructures, 10 nm wide and 1.5 nm high, whose length can reach up to several micrometres (Supplementary Fig. S1). Owing to their high aspect ratio and their internal structure composed of V_2_O_5_ sheets separated by a water layer^12^,^13^, these nanofibres are mechanically flexible, thus can easily adapt to the shape of a given template. One suitable template is ice, as the nanofibres not only are able to be dispersed in pure water, but their surface chemistry also enables taking full advantage of the structure-directing properties of the ice surface. Ice-templating is a highly versatile, low-cost synthesis method^14^,^15^,^16^ for porous materials with a hierarchic structural organization over a range of length scales. It is based on the segregation-induced templating of a second phase by a solidifying solvent, which in principle allows manufacturing any desired shape and size. Ice-templating of V_2_O_5_ nanofibres further allows generating mechanically stable functional scaffolds that feature the desired cuttlebone-like microstructure without the need of additional components or post-treatments such as sintering. The fact that this material is made of only one component facilitates the fabrication procedure and furthermore avoids problems such as functional incompatibility or ageing effects.

## Results and Discussion

**Figure 1:**
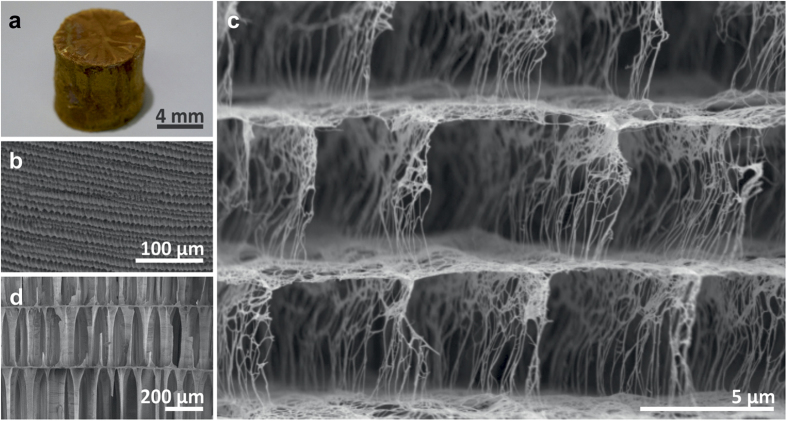
Ice-templated V_2_O_5_ nanofibre scaffold and its biological model. (**a)** Optical image of a V_2_O_5_ nanofibre scaffold with a cylindrical shape suitable for mechanical testing. (**b**) SEM image of the scaffolds exhibiting regular lamellas. (**c**) Closer inspection reveals evenly distributed interconnecting pillars. (**d**) SEM image of natural cuttlebone (*Sepia Officinalis L.*), the biological counterpart.

Ribbon-like V_2_O_5_ nanofibres were prepared by an acid-induced condensation of vanadate ions in aqueous solution^12^, using conditions that yield an average nanofibre length on the order of several micrometres (Supplementary Fig. S1). The suspension was poured into cylindrical elastomer moulds, which were then dipped into liquid nitrogen (−196 °C) to instantly freeze the solution from the walls of the moulds towards the cylinder’s centre, yielding a centrosymmetric orientation of the ice crystal plates within the macroscopic sample (Supplementary Fig. S2). The frozen cylinders were removed from the mould and subsequently freeze-dried, resulting in cylindrical samples with both diameter and height of ≈8 mm (Fig. 1a), suitable for mechanical investigations. At the microscopic scale, the samples display a highly ordered structure composed of stacked lamellas, which extend in domains of up to several hundreds of micrometres (Fig. 1b). Analogous to the ice crystal plates, the lamellas of the resulting scaffold point towards the sample’s centre (Supplementary Fig. S2). Closer inspection (Fig. 1c) highlights the vertical connection of the lamellas by regularly arranged pillars, composed of nanofibre bundles. The porous scaffold built from these bundles strikingly resembles the desired microstructure of cuttlebone with its stacked cavities assembled from aragonite fibres (Fig. 1d). It represents the first ceramic-based, close-to-ideal replica of this complex structural biomaterial, although at much smaller dimensions. Whilst cuttlebone exhibits an average lamella distance of 317.2 ± 30.9 μm and a lamella thickness of 7.0 ± 1.4 μm, the values for the V_2_O_5_ nanofibre scaffold are about two orders of magnitude smaller (5.3 ± 0.5 μm and 58.0 ± 6.0 nm, respectively), resulting in a porosity of 99.8 % that even outperforms the one of natural cuttlebone (Supplementary Table S1). The ultrahigh porosity of the synthetic scaffold is associated with two different types of pores, namely microscopic pores and pores within the walls (lamellas and pillars) of the scaffold (Fig. 1c). The latter is a distinguishing feature from the microstructure of natural cuttlebone, wherein the aragonite-based lamellas and pillars are predominantly dense (Fig. 1d).

**Figure 2:**
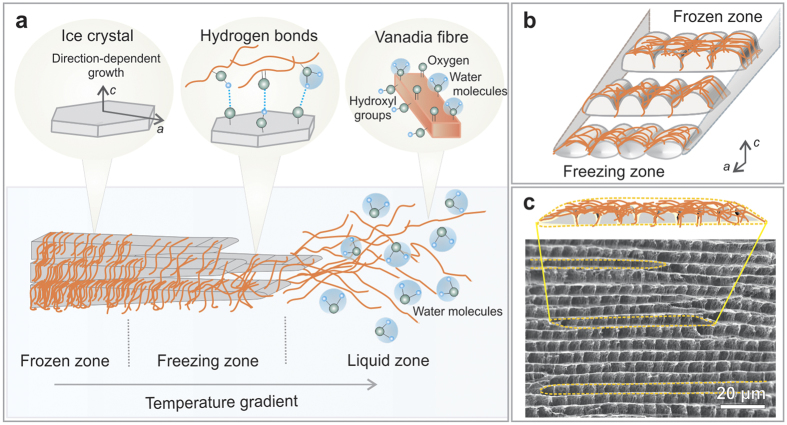
Ice-templating mechanism underlying the V_2_O_5_ nanofibre scaffold formation. (**a**) The ice crystal growth along the temperature gradient direction can be divided into three different zones. The liquid zone exhibits randomly oriented V_2_O_5_ nanofibres that form hydrogen bonds with the surrounding water molecules *via* oxygen-functionalities on the nanofibres’ surface. This functionality likewise interacts with the ice crystal surface, guiding the assembly of nanofibres in the freezing zone. The nanofibre arrangement becomes trapped and compacted, as the ice crystal front proceeds, represented by the frozen zone. The ice crystal growth is two to three orders of magnitude faster along the *a*-axis directions, as compared to the perpendicular *c*-axis direction of the plates, yielding a layered structure. Furthermore, the temperature gradient overlies the lateral expansion, leading to highly anisotropic ice crystal plates. (**b**) Arrangement and trapping of the nanofibres between the crystal tips at the front of the growing ice crystal plates creates the pillars. (**c**) Schematic illustration of the cross-section of an ice platelet in *a*-axis direction, with the fibre assembly directed by the structural features of the ice crystal plates. The SEM image shows a V_2_O_5_ nanofibre scaffold cross-section, where the replica of individual ice platelets are marked by dashed yellow lines. It can be seen that the pillars are arranged within each single replica of the ice platelet as a result of the fibres trapped within the plates.

The formation of highly ordered V_2_O_5_ nanofibre scaffolds with extreme porosity is attributed to the synergistic action of several contributing factors, as outlined in Fig. 2a. The ice-templating can be divided into three zones^17^. In the liquid zone randomly oriented nanofibres are homogeneously dispersed in the aqueous solution, stabilized by oxygen-functionalities on the nanofibres’ surfaces. The instant freezing of the aqueous nanofibre solution leads to the formation of highly anisotropic ice crystal plates oriented along the temperature gradient. The ice exists in its hexagonal modification, as revealed by high-resolution X-ray powder diffraction (XRPD) measurements on the frozen V_2_O_5_ nanofibre solution (Supplementary Fig. S3). When ice crystal plates grow inside the aqueous solution, the local fibre concentration increases and the fibres are assembled between these plates in the freezing zone. This assembly is promoted by the presence of the oxygen-containing functional groups on the fibres, which are able to participate in hydrogen bonding with such groups on adjacent fibres, and with the ice surface during the templating process. As a consequence, the fibres self-assemble into bundles, which are then able to attach as pre-organized moieties to the ice crystal surface. We have documented a similar type of assembly for paper-like V_2_O_5_ films, where the nanofibre concentration is increased by slow water evaporation^11^. The assembled fibre bundles become trapped in the frozen zone, where the temperature gradient is decreased compared to the freezing zone. The reduced temperature gradient enables the crystals to expand in the directions that are perpendicular to the gradient. This expansion is accompanied by the compacting of V_2_O_5_ nanofibre bundles between the plates, forming V_2_O_5_ nanofibre lamellas. Owing to the principal growth kinetics of hexagonal ice, the crystal thickness remains much smaller than the width and length of the ice crystal platelets (both tens to hundreds of micrometres). The growth rate in the *c*-axis direction is two orders of magnitude smaller than in the *a*-axis directions^18^. In addition, the ultrafast cooling rate in our sample fabrication amplifies the growth of the a-axis direction, which is oriented parallel to the temperature gradient, thus leads to a pronounced anisotropy towards the centre of the cylindrical sample. Based on the freezing behaviour of poly(vinyl alcohol) in water and the resulting microstructures^19^, we assume the ice growth speed to be about 375 μm/s for our freezing technique. This speed far exceeds the typical range of 1–100 μm/s, resulting in an ice crystal thickness of 5 μm, which is about one order of magnitude smaller than most previously reported ice templates^1^.

Complementary to the formation of V_2_O_5_ nanofibre lamellas between the ice crystal plates, V_2_O_5_ nanofibre pillars are formed within the plates, resulting in regular connections between the lamellas, as illustrated in Fig. 2b. X-ray computed tomography of ice crystals, growing in a colloidal silica suspension, revealed that the ice crystal plates feature a row of several small, round tips along the ice crystal front and a regular array of vertical dendrites (ridges) on the top of the crystal plates, whilst the bottom appears mostly flat^20^. As the V_2_O_5_ nanofibres are homogeneously distributed in aqueous solution, they also surround the ice crystal tips. Nanofibres that are present between two neighbouring tips get attached to the gap between the tips and form bundles, as the ice crystal front proceeds. Geometrically, the gaps between the tips exhibit an orientation parallel to the c-axis direction of the ice crystal plate, likewise the nanofibres in these gaps attach predominantly parallel to the c-axis. Upon further ice crystal growth, such parallel aligned nanofibres get trapped within the ice crystal plate. Owing to the ultrafast cooling with liquid nitrogen, the ice crystal plates’ thickness is comparable to the length of the synthesized nanofibres. The similarity in size enables the vertically oriented nanofibre bundles to be connected throughout the complete thickness of the ice crystal plates, resulting in regular bridging between the lamellas, i.e. the pillars of the freeze-dried scaffold. Figure 2c verifies that the bridging indeed happens due to trapping of nanofibres between the numerous tips of the ice crystal plates. The yellow dashed lines mark the dimensions of such ice crystal plates, revealing at least 10 pillars per plate. The pillars themselves exhibit a gradient in density (Fig. 1c). The lower part of the pillars displays rows of predominantly straight bundles, which are attached to the lamella below. Towards their top ends, they evolve into a pronounced nanofibre bundle network. This anisotropic nanofibre distribution is attributed to the presence of the dendrites on top of the ice crystal plates. The wavy top of one ice plate and the flat bottom of the adjacent ice plate lying above form small cavities, in which the nanofibres predominantly agglomerate, resulting in a higher V_2_O_5_ nanofibre concentration at the top of the pillars.

Among other ice-templated lamellar ceramic scaffolds^1^,^16^,^18^,^21^,^22^,^23^,^24^, such a subtle, highly ordered structure with regular and numerous bridging has not been reported so far, which underscores the uniqueness of the present V_2_O_5_ nanofibre scaffolds. Compared to the round or platelet-shaped ceramic particles, which in the above mentioned studies are combined with a number of additives, V_2_O_5_ nanofibres stand out due to their high aspect ratio, flexibility and oxygen-functionality, which allow them to closely adapt the structural template generated during ice crystal growth without the need of any additional components.

**Figure 3:**
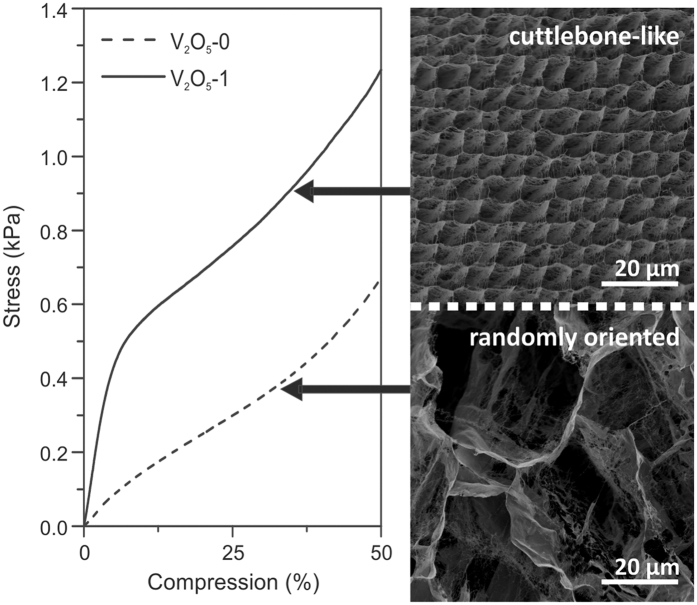
Dependence of mechanical stability on the microstructure of the ice-templated V_2_O_5_ nanofibre scaffold. The plot on the left side compares the stress-compression curves obtained for the ice-templated V_2_O_5_-1 with that of V_2_O_5_-0, the reference sample. The SEM images on the right side show that sample V_2_O_5_-1 (top image) comprises a regular, cuttlebone-like architecture, whilst sample V_2_O_5_-0 (bottom image) is much less ordered, although exhibiting the same porosity of 99.8 %.

The cuttlebone-like microstructure of the V_2_O_5_ nanofibre scaffolds was proved to be essential for achieving good mechanical stability by compressing the ice-templated scaffolds under uniaxial load up to 50 %. This compression limit was chosen to avoid sample deformation entering the densification regime^25^. The stress-compression curves in Fig. 3 signify a superior mechanical performance of the cuttlebone-like scaffold (V_2_O_5_-1), as compared to a V_2_O_5_ nanofibre scaffold, which exhibits the same ultrahigh porosity, but a random, large-pored microstructure (V_2_O_5_-0) (for preparation details see Supplementary Information). In particular, the first sample not only displays a more pronounced linear elastic regime, but also a two-fold larger stress at the same compression (Supplementary Table S2). Furthermore, the Young’s modulus of the scaffold V_2_O_5_-1 is found to be 10.73 ± 2.49 kPa, almost ten times higher than that of the reference V_2_O_5_-0 (1.73 ± 0.71 kPa). As both types of samples are prepared from the same aqueous V_2_O_5_ nanofibre solution, and also have the same porosity (99.8 %), the reason for the superior performance of the cuttlebone-like scaffold must be related to its specific architecture. It follows that the regularly arranged lamellas with almost identical distances between adjacent lamellas and neighbouring pillars in scaffold V_2_O_5_-1 can distribute the applied stress more effectively than the randomly assembled pores in the reference sample V_2_O_5_-0. Furthermore, the density of the pore walls in sample V_2_O_5_-0 is inhomogeneous and the average pore size is about one order of magnitude larger than that of the rectangular pores, which are framed by the lamellas and pillars. The small aspect ratio of these rectangular pores minimizes the lateral motion of the lamellas, resulting in an enhanced Young’s modulus.

**Figure 4:**
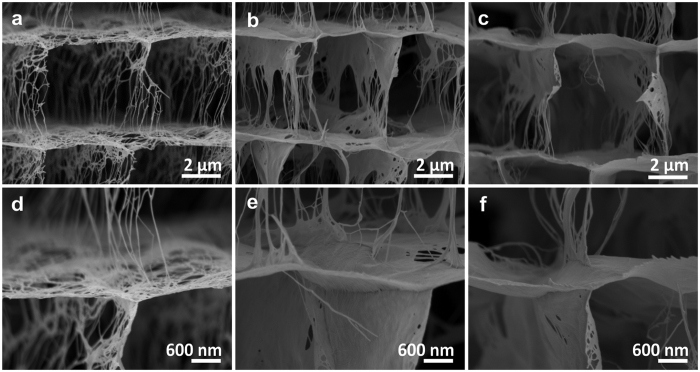
Fine-tuning the microstructure of the cuttlebone-like V_2_O_5_ nanofibre scaffold. (**a**–**c**) SEM images of the cross-section of samples prepared using V_2_O_5_ nanofibre dispersions of different concentrations, specifically (**a**) 3.5 mg/ml (sample V_2_O_5_-1), (**b**) 7.6 mg/ml (sample V_2_O_5_-2), and (**c**) 14.5 mg/ml (sample V_2_O_5_-3). (**d**–**f**) Higher resolution SEM images of the joint between a lamella and pillars of the corresponding samples.

Further tuning of the mechanical properties of this new material was achieved by reducing the wall porosity, whereby the structure of cuttlebone with its dense aragonite-based lamellas and pillars is mimicked even more closely. One simple and elegant way to control the mechanical properties along with the wall porosity is to increase the solid load of the aqueous solution in the ice-templating process (while keeping all other parameters unchanged). The effect of increasing the nanofibre concentration by respective factors of 2 (V_2_O_5_-2) and 4 (V_2_O_5_-3), as compared to the sample V_2_O_5_-1 of Fig. 1, is illustrated by the SEM images in Fig. 4a–c. Importantly, for both higher concentrations, the cuttlebone structure, including the pillars as well as the lamella distance of about 5 μm, is preserved. The latter observation is expected because the cooling rate, the crucial parameter which determines the lamella distance, was kept constant. The lamella thickness, by contrast, notably differs between the samples. It increased gradually from 58.0 ± 6.0 nm in V_2_O_5_-1 over 84.6 ± 7.7 nm in V_2_O_5_-2 to 120.0 ± 19.8 nm in V_2_O_5_-3, with increasing nanofibre concentration (Supplementary Table S1). This change is accompanied by an increase in the density of the scaffold walls, which in turn decreases the overall porosity from 99.8 % to 99.5 %. Such minute increase is to be expected, as the overall porosity is mainly governed by the volume of the micrometre-sized pores, which remains constant. The reduced wall porosity of samples prepared using a higher nanofibre concentration is nonetheless clearly detectable (Fig. 4a–c). The increased nanofibre concentration not only increases the thickness and the density of the scaffold walls, it also improves the connection between the lamellas and pillars due to a higher density of nanofibre bundles that wrap along the joint regions (Fig. 4d–f).

**Figure 5:**
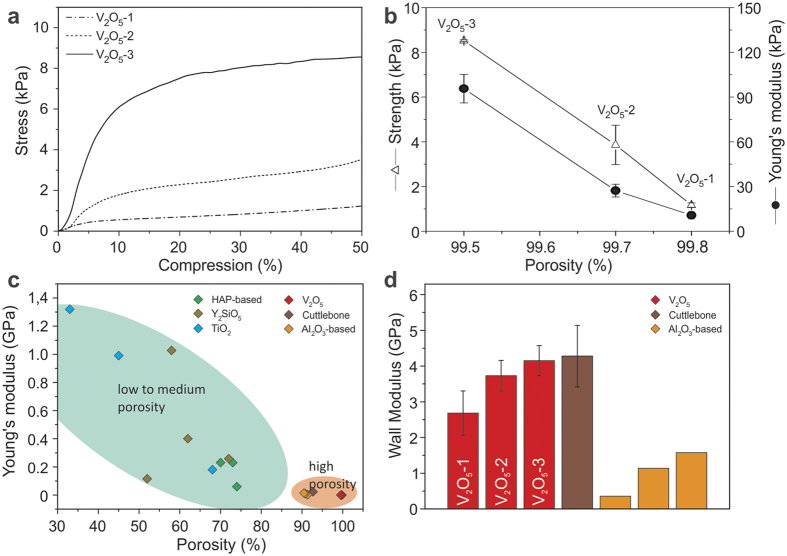
Assessment of the mechanical properties of V_2_O_5_ nanofibre scaffolds with different porosities and other lamellar ceramic scaffold. (**a**) Stress-compression curves obtained for the scaffold samples of Fig. 4 (the nanofibre concentration increases from sample V_2_O_5_-1 to V_2_O_5_-3). (**b**) Corresponding strength and Young’s modulus plotted as a function of the scaffold porosity. (**c**) Young’s modulus-porosity relationship in comparison to cuttlebone and other synthetic, lamellar ceramic scaffolds^16^,^22^,^23^,^24^. The data are grouped according to lower/medium porosity (green area) and high porosity (orange area). (**d**) Bar diagram comparing the corresponding the wall modulus of the high porosity lamellar, ceramic-based scaffolds. The error bars in b and d describe the standard deviation of the displayed values.

The structural changes upon increasing the V_2_O_5_ nanofibre concentration have an impact on the mechanical properties of the scaffolds. The behaviour of the scaffolds under compression is demonstrated by the stress-compression curves in Fig. 5a. Their shape, comprising an initial linear-elastic region followed by a deformation plateau, testifies that up to 50 % compression, all samples remain within the elastic-plastic deformation regime prior to densification. The increase in lamella thickness and wall density and improved pillar structure due to the higher nanofibre concentration manifest themselves in enhanced stiffness and strength of the porous scaffold (Supplementary Table S2), even though the sample porosity changes only slightly (Fig. 5b). It is remarkable that increasing the nanofibre concentration by a factor of 4 increases the strength as well as the Young’s modulus by almost one order of magnitude.

The porosity vs. Young’s modulus relationship of the present scaffolds is contrasted in Fig. 5c with that of cuttlebone and other lamellar ceramic-based scaffolds obtained through ice-templating^16^,^22^,^23^,^24^. In general, increasing the porosity is seen to go along with a decrease in the Young’s modulus. As the V_2_O_5_ nanofibre scaffolds exhibit by far the largest porosity among this group of lamellar scaffolds, their mechanical performance appears not to be competitive at first view. However, in order to exclude the influence of the porosity on the mechanical performance of the scaffolds, which is decisive especially in the case of highly porous scaffolds, we evaluated the wall modulus, a parameter that depends on the wall material and wall structure of the scaffold (see Supplementary Information). Based on Ashby and Medalist’s postulation of a linear relation between the relative Young’s modulus and the relative density of a cellular solid^25^, we used their equation to calculate the average Young’s modulus of the scaffold walls, thus obtaining the wall modulus (Supplementary Table S2). Comparison of the wall modulus of the three different V_2_O_5_ nanofibre scaffolds, evidences an increase from 2.68 ± 0.62 GPa (V_2_O_5_-1) to 4.15 ± 0.42 GPa (V_2_O_5_-3) (Fig. 5d). This increase is attributed to the increase in lamella thickness, the improved pillar structure and the enhanced connection between the lamellas and pillars, resulting in a stiffening of the scaffold walls. Although the lamella thickness is only up to 120.0 ± 19.8 nm (V_2_O_5_-3), the scaffold walls already approach the Young’s modulus of compact V_2_O_5_ papers (4.8 GPa), that consist of an entangled nanofibre network^26^. The scaffold V_2_O_5_-3 even approaches the wall modulus of natural cuttlebone, although the lamellas in the latter are more than one order of magnitude thicker than the V_2_O_5_ nanofibre lamellas. With respect to the Al_2_O_3_-based scaffolds^16^, the other example in the group of high porosity lamellar scaffolds, the V_2_O_5_ nanofibre scaffolds show a clearly superior wall modulus. Whilst neither type of scaffold was post-treated after the ice-templating and freeze-drying step, they nevertheless strongly differ in their composition and microstructure. This observation is explainable by the fact that the Al_2_O_3_-based scaffolds contain chitosan and gelatine, two comparably softer components, reducing the overall wall modulus. Furthermore, ice-templating of this ternary system leads to mostly inhomogeneous lamellar microstructures and a lack in cross-linking of the lamellas. However, regular ordering of the lamellas and evenly distributed pillars are crucial for obtaining excellent mechanical stability, as testified by cuttlebone and the present V_2_O_5_ nanofibre scaffolds.

In summary, the ice-templated assembly of V_2_O_5_ nanofibres provides access to a novel synthetic material whose hierarchic microstructure closely resembles that of cuttlebone, its natural counterpart. The V_2_O_5_ nanofibre scaffolds exhibit a lamellar microstructure that features regularly distributed, porous pillars, which connect adjacent nanometre-ranged thick lamellas, forming rectangular, micrometre-sized cavities. Their remarkably sophisticated lamellas bridged by regularly spaced pillars lead to an ultrahigh porosity of 99.8 %, which renders the scaffolds outstanding among similar ceramic-based porous materials. The lamellas in the latter are typically several tens to several hundreds of micrometres thick, they usually lack in crosslinking and they exhibit significantly lower porosity. Moreover, our V_2_O_5_ nanofibre scaffolds combine ultrahigh porosity with enhanced mechanical performance, which can be easily tuned by adjusting the initial V_2_O_5_ nanofibre concentration. These findings make this novel, nanostructured functional material highly interesting for applications in which the combination of large surface area, good permeability and mechanical stability is required, such as energy storage devices, sensors and catalysts.

## Methods

### Synthesis of the V_2_O_5_ nanofibres

V_2_O_5_ nanofibres were synthesized analogous to the sol-gel method developed by Livage^12^. Ammonium(meta)vanadate (1 g, Fluka) and Dowex ion exchanger (10 g, Alfa Aesar) were added to 200, 75 and 50 ml deionized water to obtain V_2_O_5_ nanofibre concentrations of 3.55, 7.64 and 19.36 mg/ml, respectively. The latter was slightly diluted with additional deionized water to be 14.51 mg/ml. Storing the mixture in an oil bath, which had a temperature of 80 °C, for 10 min while stirring initiated the nanofibre formation. The dispersion was aged for two weeks under ambient conditions, which yielded fibres with length up to several micrometres.

### Fabrication of the cuttlebone like scaffolds via ice templating

Cylindrical elastomer moulds, with a diameter of 8 mm and a height of ≈8 mm, were filled with aqueous V_2_O_5_ nanofibre solution and then dipped in liquid nitrogen to instantly freeze the solvent. The frozen cylinders were stored in a climatic chamber at −25 °C for 15 min, then de-moulded and finally placed in a pre-cooled freeze-dryer to prevent melting of the samples. Subsequent freeze-drying was performed by a P4K Peter Piatkowski freeze dryer, which first cools the chamber to −50 °C, then draws vacuum and finally slowly increases the temperature to room temperature, leading to cylindrical samples ready for mechanical characterization. For microstructural investigations, frozen samples were fractured prior to freeze-drying.

### Structural characterization

The density of the scaffolds was calculated from the sample weight and volume. The sample density divided by the scaffold’s wall density (ρ_wall_ = 2.78 g/cm^3^, as computed from the unit cell of V_2_O_5_ · nH_2_O xerogels, for which the structure was solved by Petkov *et al*^13^.) results in the scaffold’s relative density (ρ_rel_), which was then used to determine the overall porosity *via* P = (1 − ρ_rel_) × 100 (Supplementary Table S1).

SEM investigations were carried out on iridium sputtered samples (1 nm Ir) with a Zeiss Merlin SEM at 1.5 kV.

High-resolution XRPD patterns of frozen V_2_O_5_ nanofibre solutions were recorded on a laboratory powder diffractometer (D-8, Bruker, Cu-K-α_1_ radiation from primary Ge (111) Johanson monochromator; VÅNTEC-1 position sensitve detector (PSD)) in Bragg-Brentano mode equipped with a closed cycle helium cryostat (Phenix, Oxford Cryosystems). The already frozen samples (at liquid nitrogen temperature) were loaded on a precooled copper sample holder with a 4 mm deep cavity of 9 mm diameter. After evacuating the cooling chamber and adjusting the temperature to be 100 K, powder diffraction data were taken in steps of 0.016° 2Θ from 2.0 to 36.0° 2Θ within 1 hour.

### Mechanical characterization

Mechanical testing of the scaffolds was performed with a Keysight UTM 150 equipped with a CDA control unit and NanoSuite software. The system is limited to a load of 0.5 N and has a load resolution of 50 nN, enabling testing of light and highly porous samples. Samples were compressed to a maximum of 50 % with a compression rate of 8.0 · 10^−3^ mm/s. The instrument records the load and displacement, while the software automatically calculates stress, compression and the Young’s modulus. Uniaxial compression tests with a strain rate of 8.0 · 10^−3^ mm/s up to 50 % compression were also performed on rectangular samples cut out from natural cuttlebone, using a BOSE ElectroForce 3200 Series III test instrument, which is controlled *via* a WinTest7 control system and equipped with a load cell capable to bear 220 N.

## Additional Information

**How to cite this article**: Knöller, A. *et al*. Cuttlebone-like V_2_O_5_ Nanofibre Scaffolds – Advances in Structuring Cellular Solids. *Sci. Rep.* **7**, 42951; doi: 10.1038/srep42951 (2017).

**Publisher's note:** Springer Nature remains neutral with regard to jurisdictional claims in published maps and institutional affiliations.

## Supplementary Material

Supplementary Information
