# Supplementary material for: Cuttlebone-like V2O5 Nanofibre Scaffolds – Advances in Structuring Cellular Solids
Source: Sci Rep. 2017 Feb 20;7:42951. doi: 10.1038/srep42951 (PMC5317173; doi:10.1038/srep42951)
Supplement: Supplementary Information [file srep42951-s1.pdf]

Supplementary Information

Cuttlebone-like V_2O_5 Nanofibre Scaffolds – Advances in Structuring Cellular Solids

Andrea Knöller,[1] Tomče RUnčevski,[2,3] Robert E. Dinnebier,[4] Joachim Bill[1]

*and Zaklina Burghard[1,]**

[1]Institute for Materials Science, University of Stuttgart, Heisenbergstr. 3, 70569 Stuttgart, Germany
Correspondence: zaklina.burghard@imw.uni-stuttgart.de

[2]Department of Chemistry, University of California Berkeley, Berkeley, California 94720, USA.

[3]Materials Sciences Division, Lawrence Berkeley National Laboratory, Berkeley, California 94720, USA

[4]Max Planck Institute for Solid State Research, Heisenbergstr. 1, 70569 Stuttgart, Germany

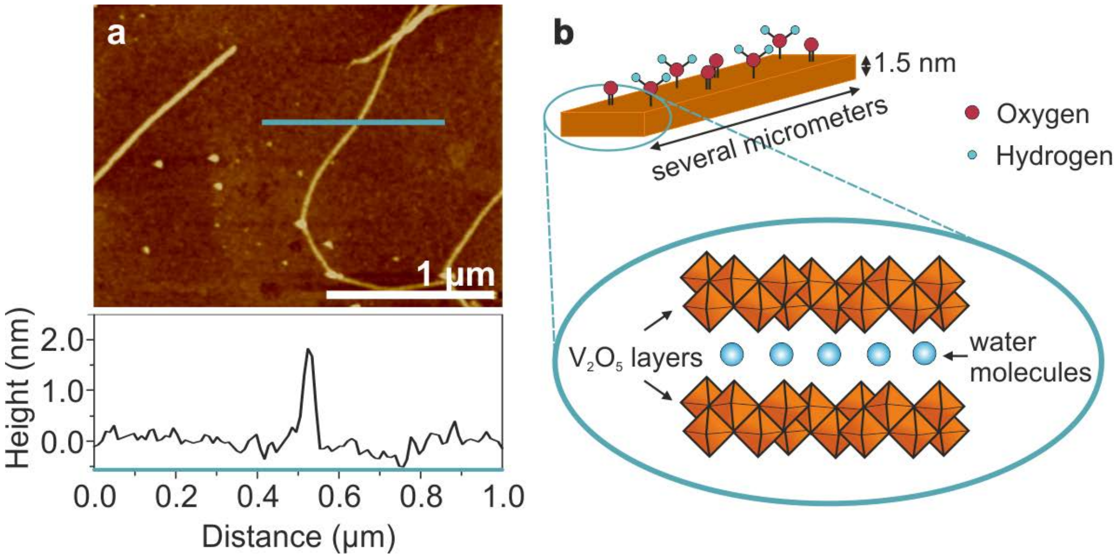

Figure S1| Sol-gel derived V_2O_5 nanofibres. a, AFM image of single V_2O_5 nanofibers with a length of several micrometres and a height of about 1.5 nm, as obtained by height profiling. **b**, Schematic depiction of a V_2O_5 nanofiber's dimension and structure. The nanofibers have a rectangular shape with a high aspect ratio and exhibit oxygen-functionalities on their surface. Furthermore, they consist of two vanadia layers with water molecules intercalated in-between.

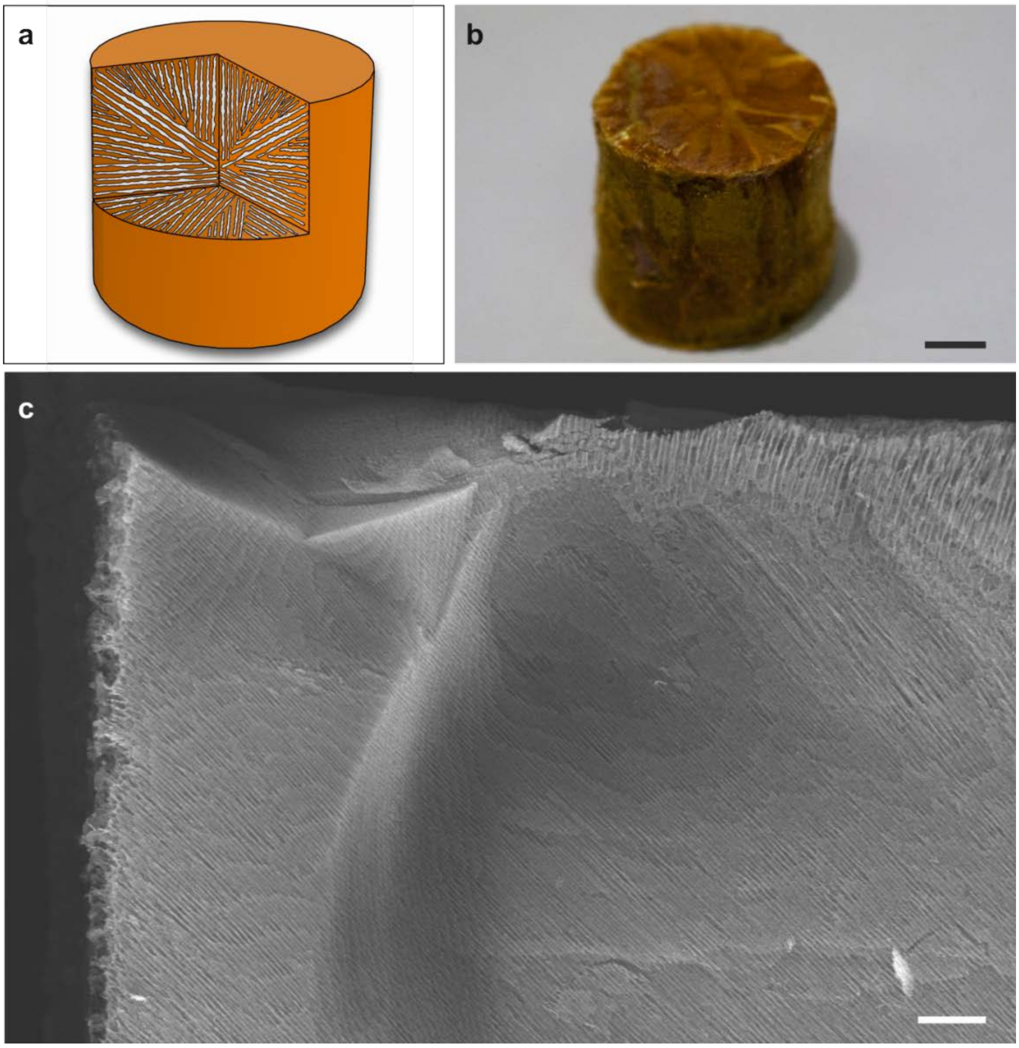

Figure S2|Lamella orientation within the cylindrical sample. a, Schematic depiction of the ice crystal plates growing from the inner wall of the mould to the cylinder's centre, leading to a centrosymmetric arrangement of lamellas within the samples. **b**, Optical image of a V_2O_5 nanofibre scaffold after freeze-drying, showing the centrosymmetric artefacts coming from the ice-templating. Scale bar: 2 mm. **c,** SEM cross-section image of a scaffold, which was fractured in frozen state before the freeze-drying step. Analogous to the scheme, the lamellas point from the samples edges toward its centre, Scale bar: 100 µm.

Table S1: Structural parameters of the V_2O_5 nanofibre scaffolds and natural cuttlebone.

Sample	V_2O_5 concentration (mg/ml)	Porosity* (%)	Lamella thickness (nm)	Lamella distance (µm)
V_2O_5-0	3.55 ± 0.04	99.8	-	-
V_2O_5-1	3.55 ± 0.04	99.8	58.0 ± 6.0	5.3 ± 0.5
V_2O_5-2	7.64 ± 0.04	99.7	84.6 ± 7.7	5.1 ± 0.5
V_2O_5-3	14.51 ± 1.36	99.5	120.0 ± 19.8	5.5 ± 0.9
cuttlebone	-	92.7	7000 ± 1387	317.2 ± 30.9

*The porosity P is defined as $P=(1-\rho_{rel})x100$. The relative density ρ_{rel} equals the scaffolds density (determined from the samples weight and volume) divided by the density of the wall material (2.78 g/cm^3), which was calculated from the unit cell of $V_2O_5 \cdot nH_2O$ xerogels, for which the structure was solved by Petkov *et al.*[1]

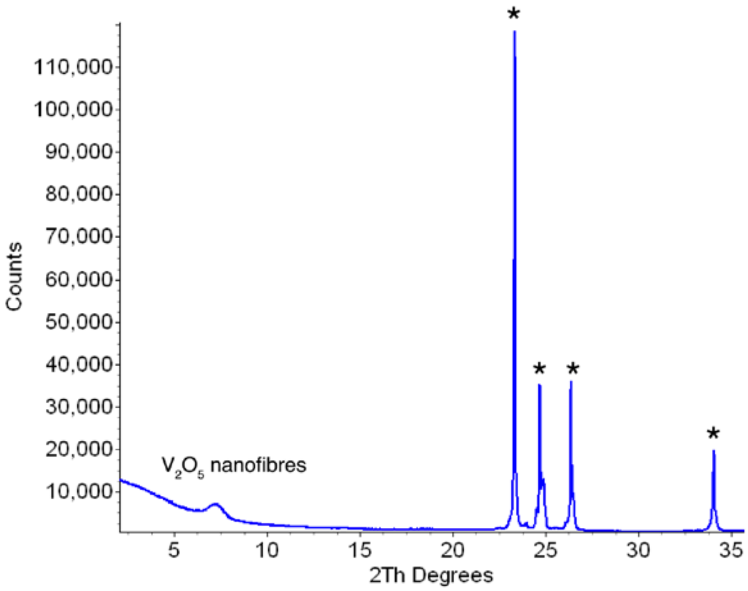

Figure S3|XRD pattern. The diffraction patterns consists of a broad hump at approx. 6.8° 2θ assigned to the V_2O_5 nanofibres and four sharp Bragg reflections (marked with *), characteristic for hexagonal ice (I_h).

Preparation of V_2O_5-0 reference sample

The reference samples were prepared using the same moulds and V_2O_5 nanofibre concentration as samples V_2O_5-1, but were slowly frozen at -25°C in a climatic chamber instead of instant freezing with liquid nitrogen. The formation of the random pore architectures can be attributed to random crystal nucleation inside the solution and much slower ice crystal growth.

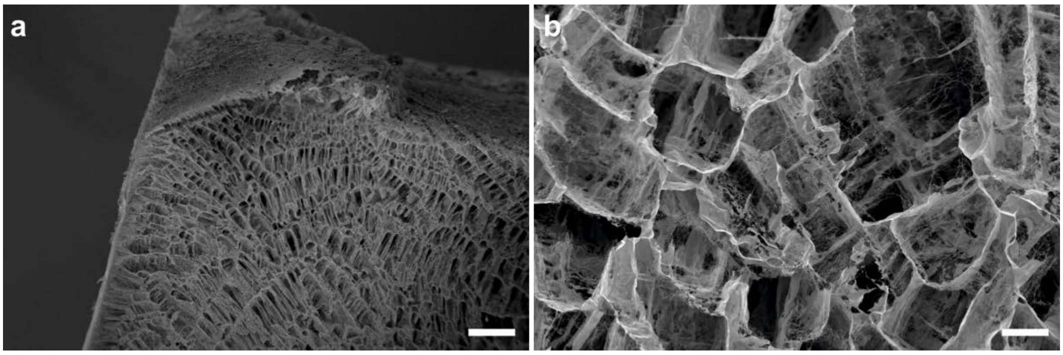

Figure S4|V_2O_5 nanofibre reference sample. a,**b**, SEM cross-section images of the reference scaffold, which was fractured in frozen state before the freeze-drying step. A freezing temperature of -25°C leads to ice crystal nucleation inside the cylinder, resulting in a randomly pored V_2O_5 nanofibre scaffold. Scale bars: (**a**) 200 µm and (**b**) 20 µm.

Mechanical characterization of Cuttlebone

Mechanical properties of cubic cuttlebone blocks (approximately 6 by 6 by 6 mm cuts from natural cuttlebone) were investigated under uniaxial compressive test. Analogous to the V_2O_5 nanofibre scaffolds, the cuttlebone was compressed up to 50 %. A typical stress-compression-curve is displayed in Fig. S4.

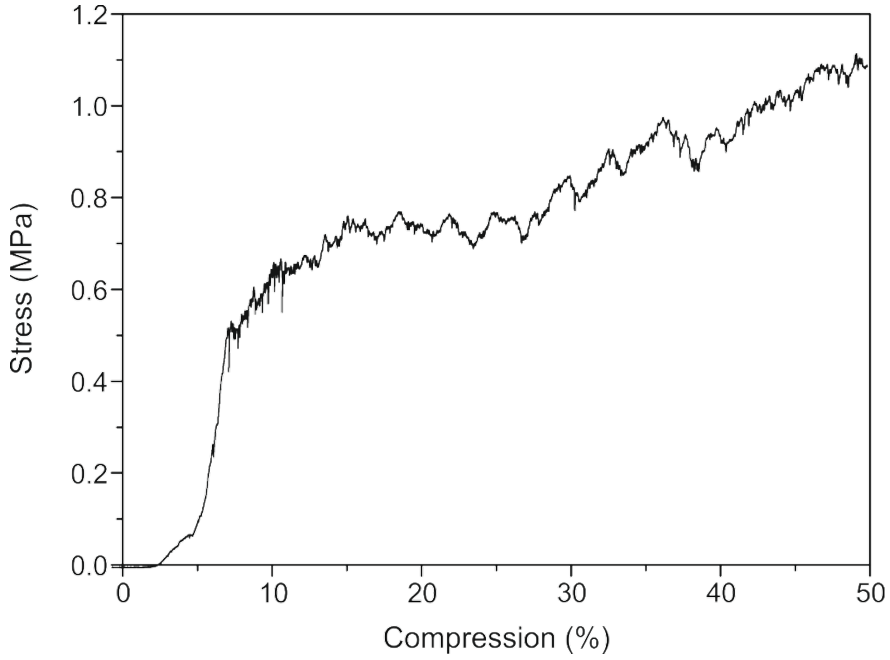

Figure S5|Mechanical testing of natural cuttlebone. Typical stress-compression curve of a rectangular sample cut from natural cuttlebone, showing a jagged trend due to brittle crushing of the lamellar scaffold.

Table S2. Mechanical properties of the V_2O_5 nanofibre scaffolds and natural cuttlebone.

Sample	Strength (kPa)	Young's modulus (kPa)	Wall Young's modulus[‡] (GPa)
V_2O_5-0	0.63 ± 0.15	1.73 ± 0.71	0.43 ± 0.18
V_2O_5-1	1.17 ± 0.10	10.73 ± 2.49	2.68 ± 0.62
V_2O_5-2	3.86 ± 1.20	27.24 ± 4.26	3.73 ± 0.43
V_2O_5-3	8.52 ± 0.04	95.66 ± 9.58	4.15 ± 0.42
cuttlebone	1171.± 125	22857 ± 4598	4.29 ± 0.86

[‡]Calculated with the equation postulated by Ashby and Medalist[2]

Calculation of the wall modulus

Predominantly independent of the class of cellular material, its relative Young's modulus correlates with its relative density via:[2]

$$\frac{E}{E_S} = C_2 \left(\frac{\rho}{\rho_S}\right)^n \qquad (1)$$

where C_2 equals 1 and n equals 2. E and ρ are the Young's modulus and density of the scaffold. E_S and ρ_S are the Young's modulus and the density of the scaffold wall, respectively.

In general, the Young's modulus of a scaffold depends not only on the type of used material and the microstructure, but also strongly on the porosity of the scaffold, leading to a much superior mechanical stability of natural cuttlebone. However, in order to exclude the influence of porosity, thus making the materials more comparable to each other, equation (1) can be applied. As the relative density (ρ/ρ_S), respectively the porosity, as well as the Young's modulus is known, the equation can be converted to calculate E_S, the wall modulus, which is in principle the Young's modulus normalized by the relative density. Thus this value

excludes the influence of the porosity on the scaffolds' mechanical performance, opening the comparison of different compositions and microstructures.

This calculation allows showing that both wall materials (aragonite fibres and V_2O_5 nanofibres) have a comparable mechanical stability. Theoretically, if the concentration of V_2O_5 nanofibres would be drastically increased, so that the porosity of the V_2O_5 nanofibre scaffolds approaches the one of natural cuttlebone, also the mechanical properties of the scaffolds would likewise approach the ones of natural cuttlebone.

References

1. Petkov, V. *et al.* Structure of V2O5·nH2O Xerogel Solved by the Atomic Pair Distribution Function Technique. *J. Am. Chem. Soc.* **124,** 10157–10162 (2002).
2. Ashby, M. F. & Medalist, R. F. M. The mechanical properties of cellular solids. *Metall. Trans. A* **14,** 1755–1769 (1983).
